# Supplementary figures and images for: The Chromosome-Scale Genome of Chitala ornata Illuminates the Evolution of Early Teleosts
Source: Biology (Basel). 2024 Jun 27;13(7):478. doi: 10.3390/biology13070478 (PMC11274187; doi:10.3390/biology13070478)

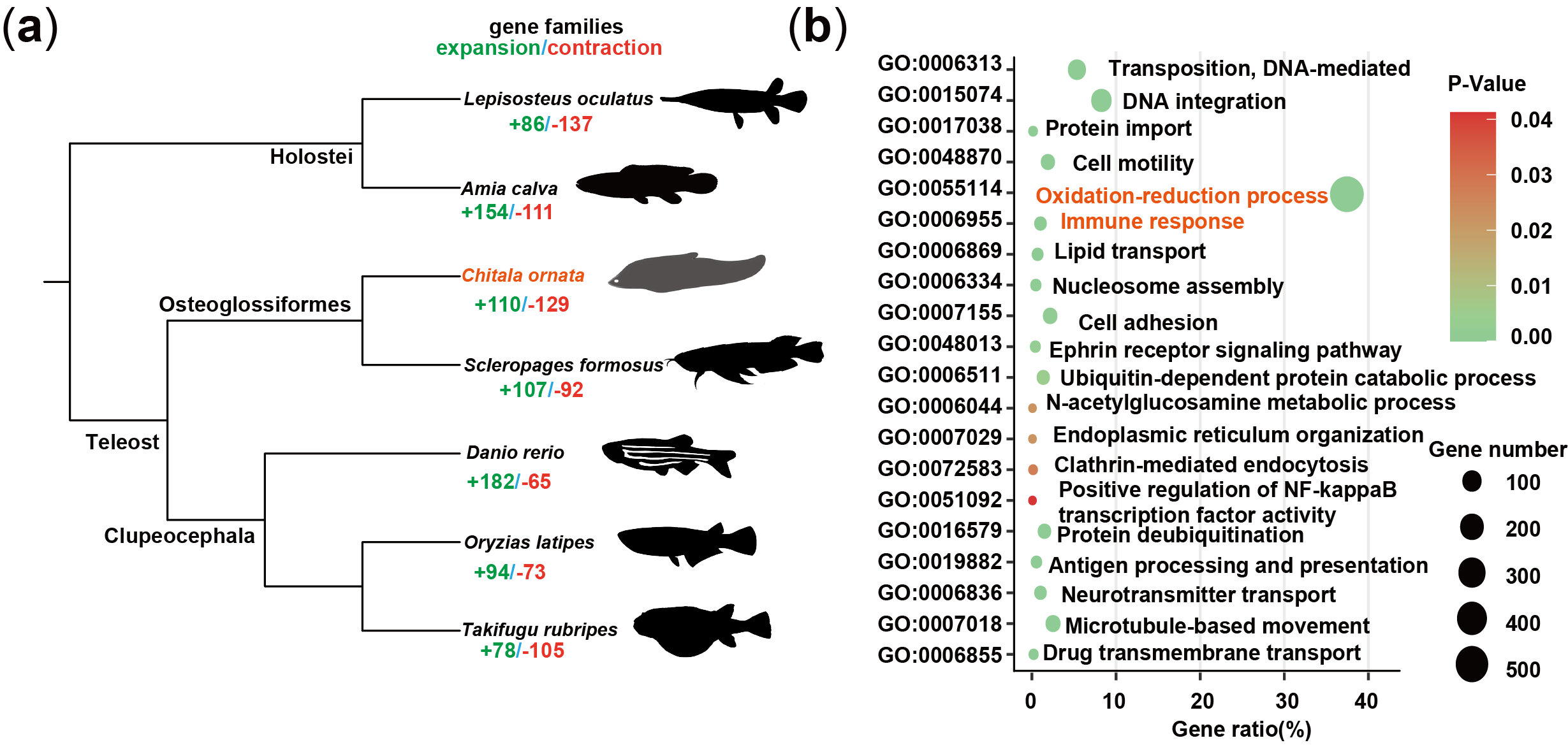

Supplement: Supplementary file 1 [file biology-13-00478-s001.zip › Figure S1.png]
